# Multigenerational Exposure to WCCo Nanomaterials—Epigenetics in the Soil Invertebrate *Enchytraeus crypticus*

**DOI:** 10.3390/nano10050836

**Published:** 2020-04-27

**Authors:** Rita C. Bicho, Janeck J. Scott-Fordsmand, Mónica J. B. Amorim

**Affiliations:** 1 Departamento de Biologia & CESAM, Universidade de Aveiro, 3810-193 Aveiro, Portugal; ritabicho@ua.pt; 2 Department of Bioscience, Aarhus University, Vejlsovej 25, PO BOX 314, DK-8600 Silkeborg, Denmark; jsf@bios.au.dk

**Keywords:** global DNA methylation, environmental species, cobalt-based nanoparticles, carbon tungsten

## Abstract

It has become clear how important it is to assess longer term effects of (nano) materials in the environment given the current evidence showing how epigenetics drives response mechanisms. Here we studied global DNA methylation in standard soil invertebrate *Enchytraeus crypticus* over 224 days when exposed to nanostructured tungsten carbide cobalt (WCCo nanomaterials (NMs)) and to cobalt (CoCl_2_) in a multigenerational experiment. In order to assess the transgenerational effect, we used a multigenerational (MG) test design consisting of four generations in spiked soil followed by two generations in clean soil. Results showed that MG exposure to WCCo NMs caused global DNA methylation to increase, which continued in unexposed generations and was associated with an increase in reproduction (phenotypic effect). In general, WCCo NMs caused more (and more consistent) methylation than CoCl_2_.

## 1. Introduction

Epigenetic mechanisms play a key role in regulating gene expression and consequently lead to phenotypic effects. Understanding how environmental stressors can affect these mechanisms will provide insight on organisms’ responses and mechanisms linked to environmental changes. This can be of particular importance for phenomena such as developmental reprogramming in early life exposures with potential multigenerational (MG) and transgenerational effects [1]. There is an increasing amount of evidence showing that nanomaterials (NMs) can cause changes in epigenetic mechanisms, e.g., in humans [2]. In light of this evidence, it has become clear how important and urgent it is to address this topic for nanomaterial risk assessment [3]. However, epigenetic studies with environmentally relevant species are very rare in the literature [4]. From the few studies reported with NMs, short-term exposure has shown epigenetic effects in plants (*Nicotiana tabacum*) exposed to aluminum oxide (Al_2_O_3_) nanoparticles (NPs) [5], in fish (*Danio rerio*) exposed to single and multi-walled carbon nanotubes (SWCNT and MWCNTs) [6] and silver (Ag) NPs [7], and in nematodes (*C. elegans*) exposed to MWCNTs [8]. However, long-term studies are among the top recommendations to assess effects of NMs [9,10,11], but research on long-term exposures covering epigenetic effects of NMs in environmental species is even more scarce. In a MG study with *C. elegans* exposed to Ag NPs [12] and another one with crustaceans (*Daphnia magna*) exposed to functionalized SWCNT-CONH_2_ [13] epigenetic changes were suggested, but this was not explicitly assessed. It is clear that, especially for invertebrates, basic knowledge on epigenetic mechanisms is lacking. For *Enchytraeus crypticus*, a standard ecotoxicological soil species, a recent publication showed an approximately 1.4% DNA methylation [14], but no methylation was observed in *Folsomia candida*, another terrestrial model invertebrate. We recently observed that copper oxide (CuO) NMs caused changes in several epigenetic mechanisms such as DNA methylation, histone modifications, and non-coding RNA in a MG study with *E. crypticus* [15]. Hence, epigenetic effects have been observed in *E. crypticus* following exposure to NMs, but no studies have dealt with nanostructured tungsten carbide cobalt (WCCo NMs). Cobalt is an essential element for most biological species, being a component of vitamin B12 and essential to processes such as DNA synthesis. Results supported that Co was also essential to *E. crypticus*. Tungsten carbide cobalt nanoparticles are used in many applications, e.g., tires and hard metal industry [16], giving the products increased wear resistance [17]. These materials are persistent; hence, it is very important to assess the long-term effects. The toxicity of WCCo particles is generally associated with an increase in activated oxygen species (AOS), given that cobalt (Co) oxidizes at the surface of tungsten carbide (WC) [18], causing proteins, lipids, and DNA damage. Despite its essentiality, Co can induce genotoxicity, inhibition of the DNA repair system, and ultimately apoptosis.

We recently performed a WCCo and CoCl_2_ MG study with *E. crypticus*, which showed effects on survival and reproduction, besides an uptake of Cobalt (Co) [19]. However, we did not measure epigenetic effects at the time. Nevertheless, the effects of Co on epigenetic mechanisms such as DNA methylation were shown in other species such as plants [20,21] and humans [22], for example, causing changes in histone modifications in human cells [23]. Further, it was also shown that cobalt-chrome NPs have genotoxicity in human cells [24,25,26] and that this genotoxicity may be caused through epigenetic mechanisms [27]. Other reports on WCCo NMs included observations of in vitro genotoxicity mostly attributed to oxidative stress [28,29,30]. Hence, there are ample indications that Co-based NMs may have epigenetic effects, and this may apply to *E. crypticus*.

The aim of this study was to evaluate global DNA methylation levels in *E. crypticus* when exposed to WCCo NMs (and CoCl_2_) in a multigenerational design, including four exposed generations plus two generations after transfer to clean soil, hence including the transgenerational effect.

## 2. Materials and Methods

### 2.1. Test Organisms

The test species *Enchytraeus crypticus* (Oligochaeta: Enchytraeidae) was used. Cultures were maintained in petri dishes with agar, please see Bicho et al. [31] for further details. Synchronized cultures were prepared, and juveniles (17–18 days) were used.

### 2.2. Test Soil

LUFA 2.2 natural standard soil (Speyer, Germany) was used. This soil is very well characterized and has a pH (0.01 M CaCl_2_) of 5.5, 1.61% organic matter, 10.0 meq/100 g CEC (cation exchange capacity), 43.3% WHC (water holding capacity), grain size distribution of 7.9% clay, 16.3% silt, and 75.8% sand as main properties.

### 2.3. Test Materials, Characterisation, and Spiking

Nanostructured tungsten carbide cobalt powder (WCCo NM) and cobalt chloride (CoCl_2_.6H_2_O, 98% purity, Sigma-Aldrich, St. Louis, MI, USA) were used. Materials were fully characterized as synthesized (Table 1) and also during exposure via a concurrent experiment. Spiking of the soil was done as reported in Ribeiro et al. [19]. In short, concentrations used correspond to the approximate 10% and 50% effect (reproduction) concentrations EC_10_ and EC_50_: 0–1200–1500 mg WCCo NMs/kg soil DW (0–100–120 mg Co/kg equivalent) and 0–110–180 mg Co/kg soil DW for CoCl_2_. WCCo NMs were spiked as dry powder following OECD (Organization for Economic Cooperation and Development) guideline [32] recommendations for dry powder non-dispersible NMs, done per individual replicate. Moisture was 50% of the maximum water holding capacity. CoCl_2_ was prepared as concentrated aqueous solution, diluted to the required concentrations, spiked onto the pre-moistened soil batches per concentration, and homogeneously mixed. The test started 1 day after spiking for both Co materials.

### 2.4. Exposure Procedure and Characterization

A multigenerational exposure test (MGt) was performed (for details see Ribeiro et al. [19]), from which organisms were sampled to analyze here. For the exposure, each replicate had 40 juveniles with 17–18 days of synchronized age. Organisms were placed in the test vessels with 40 g of soil. Each generation was exposed for a period of 32 days. The design involved six generations for CoCl_2_ and seven for WCCo NMs, during 224 and 256 days, respectively. The design included 4 + 2 (CoCl_2_) and 5 + 2 (WCCo NMs) generations (spiked soil + clean soil). Each treatment had six replicates, except for the highest concentration, which had 10 replicates to compensate for mortality. At the end of each generation, organisms from the offspring generation were used, collecting juveniles of intermediate size (n = 40) for each replicate and transferring these to the next generation. Further, for each replicate, adults (n = 20) and juveniles (n = 100) of large size were collected, snap-frozen in liquid nitrogen, and stored at −80 °C for DNA methylation analysis. For each generation, adults and juveniles were counted to assess survival and reproduction.

Cobalt (Co) concentrations were measured in soil and soil:water extracts [33] and also organisms’ bodies, using an atomic absorption spectrometer (AAS; Perkin Elmer 4100, Ueberlingen, Germany). For soil samples, 1 g dry weight was digested using 65% HNO_3_ and heated to 120 °C until all brown fumes disappeared.

Co concentrations in soil:water extracts were measured at days 0, 0.13, 1, 3, 7, 14, 21, and 28 in a concurrent experiment as described in [33]. The supernatant of a soil:water solution (1:5) was mixed for 5 min at 250 rpm (lab shaker) and then settled for 2 h. After that, 1 mL of the solution was digested using the same procedure as in soil. To measure Co concentrations in organisms, these were dried at 80 °C and then weighted. The digestion was conducted as previously described. Before the measurements, all samples were re-suspended in 2% HNO3. Results are reported in Ribeiro et al. [19].

### 2.5. Epigenetic Analysis

#### 2.5.1. DNA Extraction

For each replicate, genomic DNA was extracted from a pool of 100 juveniles. Three biological replicates were included. The procedures for the Wizard^®^ Genomic DNA Purification Kit (Promega) were followed according to the manufacturer’s protocol. DNA quantity and purity were measured (NanoDrop ND-1000 Spectrophotometer, Bath, UK).

#### 2.5.2. Global DNA Methylation

Global DNA methylation analysis was performed following the procedures described in Bicho et al. [15]. In short, measurements were done using liquid chromatography-mass spectrometry (LC-MS). Genomic DNA samples were diluted (200 ng in 10 µL of nuclease-free water), and the digestion of DNA samples into deoxyribonucleosides was performed with the addition of a digestion solution (10 µL). DNA samples were left to incubate at 37 °C for 6 h. Further, ultrapure water (80 µL) and the internal standard solution (100 µL) was added to the digested product (20 µL). Samples were injected and detected in an Agilent 6420 Triple Quadrupole LC-MS system contained in HPLC vials. Single deoxynucleosides were divided in a column Agilent ZORBAX Eclipse Plus C18 (2.1 × 100 mm, 1.8 µM). The mobile phase had the following compounds: 0.1% (*v*/*v*) formic acid in water (A) and 0.1% (*v*/*v*) formic acid in methanol (B). The gradient for the mobile phase started at 5% B, was increased to 15% B in 3 min, maintained at this composition for 1 min, and decreased to 5% B in 10 min. For the LC-MS run, the following source conditions were applied: nebulizer gas pressure fixed to 40 psi; drying gas (nitrogen) flow rate at 10 L/min, and drying gas temperature of 350 °C. Scanning of deoxynucleosides was done using a multiple reaction monitoring with a dwell time of 50 ms per compound. For the protocol validation and DNA sample quantification, the mass transitions of compounds were 5mdC 242.1/126.1, D3mdC 245.1/129.1, dG 268.1/152.2, and C10N5dG 282/162.2. The collision energy was of 8 V for 5mdC and D3mdC, whereas for dG and C10N5dG it was of 4 V. The standard curve included increasing amounts of 5mdC (0–2.07 µM, 0–3%) to a fixed amount of G (345 nM). The software Agilent MassHunter Quantitative Data Analysis (Agilent, Santa Clara, CA, USA) was used. 

### 2.6. Data Analysis

For the results on global DNA methylation, t-test and two-way analysis of variance (ANOVA) (*p* ≤ 0.05) were performed, the two independent variables were concentration and exposure time (Fx generation) for the significance of variables and their interaction [34]. A linear regression analysis was used between methylation and reproduction data [34].

## 3. Results

**Figure 1:**
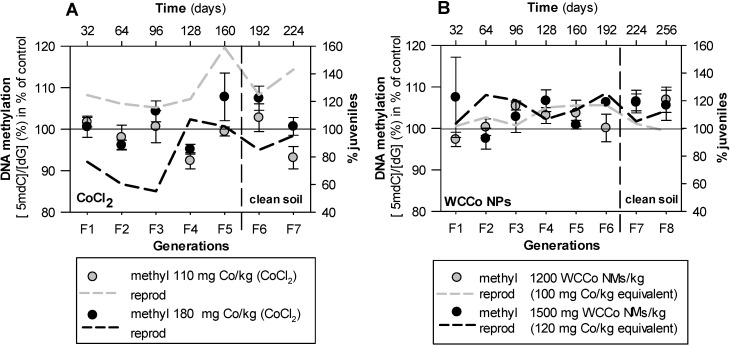
Global DNA methylation (LC-MS, Liquid Chromatography—Mass Spectrometry measurements) for *Enchytraeus crypticus* after multigenerational exposure to the reproduction EC_10_ and EC_50_ (and control samples) of (**A**) CoCl_2_ (0–110–180 mg Co/kg soil DW) and (**B**) WCCo NMs (0–1200–1500 mg WCCo NMs/kg soil DW, 0–100–120 mg Co/kg equivalent) in LUFA 2.2 soil. All values are expressed as average ± standard error (Av ± SE).

Results from global DNA methylation across multiple generations (Figure 1) showed no significant differences between effects of concentration, exposure time (generation), or the interaction between the two factors (two-way ANOVA). For WCCo NMs exposure, there was an increase in DNA methylation from F1 to F3 (for the EC10 (*p* = 0.04) and EC50 (*p* = 0.006), an outlier in F1 was removed), after which it remained generally elevated, including after transfer to clean soil. For the F3 to F8 period there was a significant (*p* = 0.03) linear regression between DNA methylation and number of juveniles for EC10 (juveniles = −2.90 DNA-Methylation + 411; R^2^ = 0.76). The CoCl_2_ results presented higher variability and no clear pattern.

Results on the apical endpoints survival and reproduction (dashed plotted lines, Figure 1) as well as measurements of internal Co body concentrations in *E. crypticus* were previously reported in Ribeiro et al. [19].

## 4. Discussion

In summary, effects at the organism level were observed on survival for CoCl_2_ but not for WCCo. Cobalt caused a significant decrease in reproduction at 180 mg Co/kg soil Dry Weight (D.W.) (ca. EC50) [33], and this toxicity rapidly increased to lethal levels thereafter (LC50 = 260 mg Co/kg soil D.W.). The analysis of CoCl_2_ spiked soil showed relatively higher Co in solution in higher concentrations, i.e., more Co than the equivalent relative difference between concentrations [33], which explains the observed survival and reproduction effects. Cobalt is also well-known as an oxidative stress agent; hence, the effects (survival and reproduction) could indicate the irreversible impairment of antioxidant strategies this for higher concentrations. For WCCo, toxicity was shown to be due to a combined effect between WC and Co, and the impact was clearly observed with increased exposure time, i.e., with twice the standard exposure period (56 days instead of 28). To assess the impact along generations, the exposure was obviously at sub-lethal concentrations. Reproduction increased at lower concentrations of Co. Internal body measurements showed Co uptake from exposure to both CoCl_2_ and WCCo, but no toxicity occurred for WCCo; furthermore, Co from CoCl_2_ exposure was apparently stored, while for WCCo it was eliminated [19]. Results for the MG exposure to WCCo NMs showed an increase in reproduction along generations. A similar pattern was observed for CoCl_2_ [19].

For WCCo NMs multigenerational exposure, the increased DNA methylation from F3 was inversely correlated with effects at reproduction level at the EC10 level. Since this increase methylation level continued in the clean media generations, this indicates a transgenerational effect. Although this is significant, this observation obviously calls for further studies, partly to reconfirm this and also to identify the mechanism behind, e.g., what exact mechanism and specific methylation causes this change. We also assessed whether there was a better correlation between the DNA methylation of the Fn with the juvenile number of the Fn^+1^ generation, to see if methylation mainly caused an effect on the subsequent generation, but this was not the case. Bicho et al. [15], who studied the MG effects of CuO NMs exposure also observed a pattern of increase for global DNA methylation levels, which corresponded with the phenotypic effects on reproduction, further indicating transgenerational effects. Epigenetic changes can lead to stress adaptation with consequences for population dynamics [35,36], causing a change in populations. We observed no similarity in the methylation pattern between the CoCl_2_ exposure and the WCCo NMs, possibly due to a difference in induction mechanisms by the two materials or because Co uptake patterns were different. Cobalt bioaccumulated in the CoCl_2_ [33] exposure. Cobalt is an essential element, and this could explain the increase in reproduction for the lowest CoCl_2_ exposure concentration, hence its beneficial effects at lower concentrations [19]. For WCCo NMs, this is much less clear. Uptake studies for CoCl_2_ indicated that it was bioaccumulated and apparently stored, as levels remained stable (ca. 120 mg Co/kg body D.W.) and high even after transfer to clean soil [19]. For WCCo NMs exposure, Co accumulation increased along generations up until F3, after which it started to decrease, similar to a detoxification activation mechanism process, with a complete elimination when transferred to clean soil. The observed decrease in the impact on reproduction could be associated with the observed epigenetic shift. Whether the activated mechanisms were led by epigenetics remains uncertain and requires further evidence for CoCl_2_; however, for WCCo NMs, the epigenetic change remained in the absence of Co and was hence transferred between generations.

Hence, there is an indication that WCCo NMs in general cause more (and more consistent) methylation than CoCl_2_. There was an increase after the F3 generation; although a linear correlation was observed at the EC10 level, there was not a clear 1:1 correlation between methylation and reproduction. Most studies done are in vertebrates, for instance, a correlation has been shown between reproductive history and DNA methylation in humans [37]. However, for invertebrates, studies are very scarce; to our knowledge, the present study and the one performed with exposure to CuO NMs [15] are so far the only studies providing evidence of epigenetics (global DNA methylation) linked to impact on reproduction.

In summary, our results support the importance of assessing longer-term effects of (nano) materials in the environment. Here we studied global DNA methylation in standard soil invertebrate *Enchytraeus crypticus* exposed to WCCo nanomaterials and to cobalt (CoCl_2_) over multiple generations, showing that MG exposure to WCCo NMs caused global DNA methylation to increase, which continued in unexposed generations and was associated with an increase in reproduction (phenotypic effect). In general, WCCo NMs caused more (and more consistent) methylation than CoCl_2_. The next interesting steps will obviously involve identifying precisely where on the genome such methylation takes place and at which exact life cycle stage(s) this happens. Further, it seems obvious that such a methylation does not only apply to this species. Hence, studies with other species are also required, especially because from a risk assessment perspective, we wish to know whether this is a general phenomenon potentially affecting many invertebrate species.

## Figures and Tables

**Table 1:** Characterization of tungsten carbide cobalt nanoparticles (WCCo NPs) (FP7 SUN: Sustainable Nanotechnologies). TEM: transmission electron microscope; XRD: X-ray diffraction; DLS: dynamic light scattering; BET: Brunauer, Emmett, and Teller; ELS: electrophoretic light scattering; FTIR: Fourier-transform infrared spectroscopy; XPS: X-ray photoelectron spectroscopy.

Characteristics	WCCo NP	Technique
**Source**	NBM Nanomaterialia, Italy	
**Composition (%)**	Tungsten carbide (WC < 88% Wt., CAS 12070-12-1) Cobalt (Co = 8.32% Wt., CAS 744-48-4)	ICP-MS
**Primary Size distribution [nm] Average (Min-Max) Mode [nm] (1st and 3rd quartile)**	170 (23-1446)48 (69; 280)	TEM
**Crystalline size (Average) [nm]**	15.4	XRD
**Iso Electric Point (pH)**	<2	pH
**Dispersability in water: D50 [nm]; Average Agglomeration Number (AAN)**	182.8 ± 21.5; 31	DLS
**Specific Surface Area [m^2^.g ^−1^]**	6.6 ± 0.4	BET
**Z-potential [mV]**	7.1 ± 0.5	ELS
**Structure**	O-W-O	FTIR and/or RAMAN
**Pore size [nm]**	Non-porous	BET
**Surface Chemistry [atomic fraction]**	Co = 0.08 ± 0.01	XPS
	W = 0.05 ± 0.01	
	O = 0.31 ± 0.03	
	C = 0.56 ± 0.05	
